# Response to a commentary “Comment on “Collateral damage: Cardiovascular and respiratory implications of tear gas deployment during peaceful protest”: Caution in interpreting the reported associations”

**DOI:** 10.1016/j.toxrep.2025.102198

**Published:** 2026-01-02

**Authors:** Konstantine Chakhunashvili, Davit G. Chakhunashvili

**Affiliations:** Caucasus University, Tbilisi, Georgia; Alte University, International School of Medicine, Tbilisi, Georgia

This is response to a Kandelaki et al. „Comment on “Collateral damage: Cardiovascular and respiratory implications of tear gas deployment during peaceful protest”: Caution in interpreting the reported associations”[1]. It is of vital importance to mention that the author fails to mention the new circumstances including BBC eye investigation, participants of the movie being called in for questioning, along with the authors of the study, and including the participants of the study who publicly mentioned that they took part in the study [2].

Regarding the study design, this investigation followed a case–control framework, in which cases were individuals who developed new symptoms during the protests (also were exposed to the chemical agents), while controls were individuals who did not develop symptoms (also were not exposed to the agents). Exposure to tear gas occurred during the protest period and was assessed in both groups. Although odds ratios (ORs) were not originally reported in the manuscript, we report them here. Right bundle branch block (RBBB) was associated with higher odds among exposed individuals (OR = 7.25, 95 % CI [1.59, 33.08], *p* = .010), as was T-wave inversion (OR = 12.24, 95 % CI [1.56, 95.99], *p* = .017). Sclerodermal changes showed an elevated odds ratio that did not reach conventional statistical significance (OR = 16.89, 95 % CI [0.88, 325.04], *p* = .061).

Regarding statistical considerations and post hoc analyses, we acknowledge that post hoc power calculations are inherently dependent on observed effect sizes and do not substitute for a priori sample size determination. Nevertheless, in observational studies where sample size is constrained by feasibility, ethical, or political considerations (such as above mentioned possibility of legal harassment from the authorities), post hoc power assessment may provide contextual insight into the interpretation of both significant and non-significant finding. Bonferroni correction for multiple testing (m = 3) shows that under this correction, associations for right bundle branch block (p = .005) and T-wave inversion (p = .012) remained statistically significant [2]. Together with the magnitude of the observed odds ratios, this suggests that the statistically significant findings are robust to conservative correction, while the limited sample size primarily increases the risk of Type II error for outcomes that did not reach statistical significance, rather than inflation of observed effects. Also, it needs to be mentioned that type II error does not at all apply to overestimation or inflation of the results but detection of false negatives textbook definition: A Type II error occurs when a real difference exists but is not detected by the study [3], while type M and Type II error definitions when compared are: Type II error concerns missed true effects, whereas overestimation of statistically significant effects is described by Type M (magnitude) error (Gelman and Carlin, 2014)” [4]. Since, the authors mention type II error either they do not know the difference or purposefully make inaccurate statement about type II error and overestimation.

In terms of the control group it has been mentioned that the participants had been matched for the key demographic variables (mean age, biologic sex, smoking), however, we should add that they were recruited via 1 on 1 communication and not via survey. In terms of temporal control – specifically baseline – baseline tests could not have been done in a real world scenario, that would require in advance knowledge that there was going to be a dispersal and use of special means. Longitudinal data would indeed be necessary which is mentioned in the original paper along with other limitations mentioned .

The commentary mentions that the ECG changes could be attributed to various socio-economic, geographic or political data, which does not explain why the control was not affected.

In summary, the commentary by Kandelaki et al. contains substantial factual, methodological, and statistical inaccuracies that materially misrepresent both the design and the findings of the original study. The author fails to acknowledge critical contextual developments surrounding the investigation, mischaracterize the case–control framework, and advance claims regarding effect inflation and statistical validity that are not supported by the data. Independent calculation of effect estimates demonstrates that the principal findings—particularly right bundle branch block and T-wave inversion—remain statistically significant even under conservative multiple-testing correction, directly contradicting the commentary’s assertions. Moreover, the discussion of Type II error reflects a fundamental misunderstanding of established statistical definitions, conflating false-negative risk with effect overestimation.

Speculative explanations invoking unmeasured socio-economic or political factors are presented without evidentiary support and fail to account for the absence of similar findings in the matched, unexposed control group. Methodological critiques regarding baseline measurements and recruitment procedures similarly disregard the practical constraints inherent to real-world observational research conducted during unforeseen protest events. Collectively, these issues indicate not a difference in interpretation, but a pattern of incorrect statements and unsupported conclusions that undermine the scientific integrity of the commentary. Especially in light of the fact that in conclusion and limitation section we mention that more studies are needed and the limitations prompt us to interpete results with caution [5].

Given the cumulative weight of these errors, their direct impact on the interpretation of the original findings, and the potential to mislead readers regarding both statistical principles and real-world toxicological evidence, we conclude that the commentary does not meet the standards.

## Authorship

All named authors meet the International Committee of Medical Journal Editors (ICMJE) criteria for authorship for this article, take responsibility for the integrity of the work as a whole, and have given their approval for this version to be published.

## CRediT authorship contribution statement

**Davit G. Chakhunashvili:** Writing – original draft, Writing – review & editing. **Konstantine Chakhunashvili:** Conceptualization, Writing – original draft, Writing – review & editing, Formal analysis.

## Ethics approval and consent to participate

N/A.

## Consent for publication

N/A.

## Clinical Trial Number

Not applicable.

## Funding

No financial assistance was received to support the study.

## Competing interests

There are no potential conflicts of interest.

## Declaration of Competing Interest

The authors declare that they have no known competing financial interests or personal relationships that could have appeared to influence the work reported in this paper.

## Data Availability

Data will be made available on request.
